# Brainstem response patterns in deeply-sedated critically-ill patients predict 28-day mortality

**DOI:** 10.1371/journal.pone.0176012

**Published:** 2017-04-25

**Authors:** Benjamin Rohaut, Raphael Porcher, Tarik Hissem, Nicholas Heming, Patrick Chillet, Kamel Djedaini, Guy Moneger, Stanislas Kandelman, Jeremy Allary, Alain Cariou, Romain Sonneville, Andréa Polito, Marion Antona, Eric Azabou, Djillali Annane, Shidasp Siami, Fabrice Chrétien, Jean Mantz, Tarek Sharshar, Groupe d’Exploration Neurologique en Réanimation (GENER)

**Affiliations:** 1 Neurological Departement Intensive Care Unit, Assistance Publique - Hôpitaux de Paris (AP-HP), Pitié-Salpétrière Hospital, Paris, France; 2 Sorbonne University, UPMC Univ Paris 06, Faculté de Médecine Pitié-Salpêtrière, Paris, France; 3 Institut du Cerveau et de la Moelle épinière, ICM, PICNIC Lab, Paris, France; 4 INSERM, U 1127, Paris, France; 5 Center for Clinical Epidemiology, AP-HP, Hôtel Dieu Hospital, Descartes University, Paris, France; 6 General Intensive Care Unit, Sud Essonne Hospital, Etampes, France; 7 General Intensive Care Unit, AP-HP, Raymond Poincaré Hospital, University of Versailles Saint-Quentin en Yvelines, Garches, France; 8 General Intensive Care Unit, Chalons en Champagne Hospital, Chalons en Champagne, France; 9 General Intensive Care Unit, Geoffroy Saint-Hilaire Hospital, Paris France; 10 Department of Anesthesiology and Intensive Care Unit, AP-HP, Beaujon–Claude Bernard Hospital, Diderot University, Paris, France; 11 Intensive Care Unit, AP-HP, Cochin Hospital, Descartes University, Paris, France; 12 Medical Intensive Care Unit, AP-HP, Bichat–Claude Bernard Hospital, Diderot University, Paris, France; 13 Surgical Intensive Care Unit, AP-HP, Cochin Hospital, Descartes University, Paris, France; 14 Department of Physiology, AP-HP, Raymond Poincaré Hospital, University of Versailles Saint-Quentin en Yvelines, Garches, France; 15 Laboratory of Human Histopathology and Animal Models, Pasteur Institut, Paris, France; 16 Department of Neuropathology, Saint Anne Hospital, Descartes University, Paris, France; 17 Department of Anesthesiology and Intensive Care Unit, AP-HP, Georges Pompidou European Hospital, Descartes University, Paris, France; University of Colorado Denver, UNITED STATES

## Abstract

**Background and purpose:**

Deep sedation is associated with acute brain dysfunction and increased mortality. We had previously shown that early-assessed brainstem reflexes may predict outcome in deeply sedated patients. The primary objective was to determine whether patterns of brainstem reflexes might predict mortality in deeply sedated patients. The secondary objective was to generate a score predicting mortality in these patients.

**Methods:**

Observational prospective multicenter cohort study of 148 non-brain injured deeply sedated patients, defined by a Richmond Assessment sedation Scale (RASS) <-3. Brainstem reflexes and Glasgow Coma Scale were assessed within 24 hours of sedation and categorized using latent class analysis. The Full Outline Of Unresponsiveness score (FOUR) was also assessed. Primary outcome measure was 28-day mortality. A “Brainstem Responses Assessment Sedation Score” (BRASS) was generated.

**Results:**

Two distinct sub-phenotypes referred as homogeneous and heterogeneous brainstem reactivity were identified (accounting for respectively 54.6% and 45.4% of patients). Homogeneous brainstem reactivity was characterized by preserved reactivity to nociceptive stimuli and a partial and topographically homogenous depression of brainstem reflexes. Heterogeneous brainstem reactivity was characterized by a loss of reactivity to nociceptive stimuli associated with heterogeneous brainstem reflexes depression. Heterogeneous sub-phenotype was a predictor of increased risk of 28-day mortality after adjustment to Simplified Acute Physiology Score-II (SAPS-II) and RASS (Odds Ratio [95% confidence interval] = 6.44 [2.63–15.8]; p<0.0001) or Sequential Organ Failure Assessment (SOFA) and RASS (OR [95%CI] = 5.02 [2.01–12.5]; p = 0.0005). The BRASS (and marginally the FOUR) predicted 28-day mortality (c-index [95%CI] = 0.69 [0.54–0.84] and 0.65 [0.49–0.80] respectively).

**Conclusion:**

In this prospective cohort study, around half of all deeply sedated critically ill patients displayed an early particular neurological sub-phenotype predicting 28-day mortality, which may reflect a dysfunction of the brainstem.

## Introduction

Current guidelines recommend that sedation be monitored, titrated and discontinued as soon as possible and that benzodiazepines be avoided in critically ill patients [1,2]. However, deep sedation, defined by a Richmond Assessment Sedation Scale (RASS) below -3, may be required in a handful of conditions, including severe respiratory failure and septic shock [1,3]. Indeed, deep sedation is administered in 30 to 70% of all ICU patients [4–8], and benzodiazepines remain the most commonly used sedative in Intensive Care Units (ICU; [6]). Deep sedation is associated with delayed awakening, increased mortality [4,6,9], acute brain dysfunction [10–12] and a long-term impact on mental health [13]. The mechanisms linking deep sedation to mortality and delirium remain unclear. Here, we hypothesized that brainstem dysfunction might be involved, as early abolition of the cough and oculocephalic reflexes is associated with mortality and delirium [14]. In order to further improve our understanding of the interaction between sedation, critical illness and brainstem reflexes, we sought to define particular neurological sub-phenotypes in non-brain injured, deeply sedated, critically ill patients.

The primary objective of this study was to determine whether a particular pattern of brainstem responses predicted 28-day mortality in deeply sedated, critically ill patients; the secondary objective was to generate and validate a prognostic score of mortality in these patients, based on clinical examination of brainstem reflexes.

## Materials and methods

### Study design and setting

This was a prospective, multicenter, observational study, approved by the ethics committee of Saint-Germain-en-Laye, France (n°05043). Due to the observational nature of the study, the need for written informed consent was waived. Patients were recruited in four medico-surgical and two surgical ICUs. Inclusions took place over three distinct periods: between December 2007 and June 2009, between November 2011 and August 2012 and between December 2012 and August 2014. We reasoned that repeating short periods of inclusion would optimize patients’ recruitment. Forty-six patients enrolled over the first period (from 2007 to 2009) were derived from the validation set of a previously described study, and included all patients with a RASS<-3 [14]. The Strengthening the Reporting of Observational Studies in Epidemiology (STROBE) guidelines were followed thoroughly [15].

### Participants

Eligible patients were invasively mechanically ventilated and required continuous intravenous sedation by midazolam alone or in association with sufentanil for at least 12 hrs. All participants were deeply sedated, defined by a RASS<-3, because of high discomfort, threatening agitation, sever ventilator asynchrony or severely impaired gas exchange. Patients were excluded if they were treated by neuromuscular blocking agents, were affected by a peripheral neurologic disorder involving the cranial nerves or had been referred to the ICU for a stroke, central nervous system infection, neuro-inflammatory disease or traumatic brain injury.

### Sedation

Decisions to initiate, adjust and discontinue sedation were taken by the physicians in charge. Depth of sedation was assessed every four hours using the RASS [3]. Onset time and the reason for initiating sedation were recorded. The cumulative administered doses of midazolam and sufentanil were expressed as milligrams/kilogram and micrograms/kilogram. Discontinuation of sedation occurred through daily interruption and/or titration. Discontinuation was decided every morning. Titration was monitored at least twice daily using the RASS.

### Neurologic examination

The detailed procedure pertaining to neurological examination is described elsewhere [14]. Briefly, we assessed: 1) the level of consciousness prior to intubation and initiation of sedation, using the Glasgow Coma Scale (GCS); 2) the depth of sedation using the RASS; 3) reactivity, using the motor and eye response components of the GCS; 3) the brainstem reflexes, including pupil size (miosis, normal, or mydriasis), pupillary light reflex, corneal reflex, grimace in response to a bilateral and strong pressure to the retro-mandibular region, oculocephalic reflex (OCR) to lateral passive head rotation, and cough reflex in response to tracheal suctioning. Reactivity and brainstem reflexes were scored as present or abolished (see S1 Appendix). Additionally, the Full Outline Of Unresponsiveness (FOUR)-score was assessed [16]. Neurologic examination was performed following 24 ± 12h hours of the initiation of sedation.

### Baseline clinical and biological data

Demographic characteristics, body weight, the date, time, cause and category (medical or surgical) of ICU admission, co-morbidities, risk factors for delirium using the Pre-Deleric Score [17,18], date, time and cause of invasive mechanical ventilation initiation were recorded. The Simplified Acute Physiological Score II (SAPS-II; [19]), the Sequential Organ Failure Assessment (SOFA; [20]), as well as key interventions and standard biological tests needed to calculate these scores were recorded. The results of neurological examination on day 4 in patients with RASS<-3, of brain imaging and of somatosensory and brainstem auditory evoked potentials (SSEP and BAEP), when available, were also recorded. The SSEP medullo-cortical (P14-N20) and BAEP ponto-mesencephalic (III-V) conduction times were collected and considered delayed when the mean value of the left and right side was greater than the mean +2.5 SD of the inter-latencies obtained in 20 healthy subjects (>4.6 ms and >2.2 ms respectively). Clinical, biological and brain imaging (CT and/or MRI) data were collected as part of the routine care, evoked potentials originated from a preliminary neurophysiological study.

### Outcomes

Primary outcome was the 28-day mortality. The date and cause of death were collected. Secondary outcome was altered mental status following discontinuation of sedation, defined by the occurrence of delayed awakening and/or delirium. Delayed awakening and delirium after discontinuation of sedation were assessed daily using the RASS and the confusion assessment method for the ICU (CAM-ICU; [21]), respectively. Delayed awakening was defined by RASS<-1 after 3 days of discontinuation of sedation. The duration of mechanical ventilation and the length of stay in the ICU were also collected.

### Bias and confounding factors

We sought to mitigate potential confounding factors that may have influenced neurological examination and relevant outcomes. Neurological examination was performed by a specifically trained senior ICU physician. Inter-observer agreement for neurological examination was previously shown to be satisfactory (kappa scores ranged from 0.62 to 1; [14]. Management of sedation was assessed by collecting the duration and the modalities of discontinuation of sedation and RASS. The cause of death and its main risk factors were collected, including severity scores (SAPS-II and daily SOFA) and the cause of critical illness. In this manner, we were able to compare groups by study period, ensuring that the management of sedation was appropriate and that the selected population was representative of severe clinical situations where patient could receive deep sedation.

### Statistical analysis

#### Latent class analysis (LCA)

Patients were classified into clinical sub-phenotypes using latent class analysis (LCA). LCA is a statistical model that aims at identifying unobservable subgroups within a population. It posits that there is an underlying unobserved categorical variable that divides the population into a certain number of exclusive groups, called latent classes. LCA thus aims at inferring the number of classes and the class membership of individuals from a set of measured variables [22,23] No outcome parameter was included in the LCA. Brainstem reflexes assessed on day 1 were incorporated into this analysis as dichotomous variables (present or absent). GCS eye and motor sub-scores were also taken into account, after categorization as a binary variable (1 vs >1).

The optimum number of latent classes was determined by looking at the log-likelihood, the Akaike information criterion (AIC), the Bayesian information criterion (BIC) as well as goodness-of-fit statistics (Pearson chi-square, likelihood-ratio chi-square, G-square and bootstrap likelihood ratio test (BLRT); S1 Table). In case of disagreement between the various indexes, we gave priority to the BIC. Once a model was selected, patients were assigned their most likely class to provide an exploratory analysis of differences between the latent classes, thereafter denoted sub-phenotypes.

Patient characteristics were compared between sub-phenotypes using Fisher’s exact tests and Wilcoxon rank-sum tests. Association with 28-day mortality was assessed after adjusting for the SAPS-II or the SOFA (with and without the RASS) using logistic regression models, which were compared to similar models without the LCA sub-phenotype.

#### Construction of the "Brainstem Responses Assessment Sedation score" (BRASS)

Using the same variables as used for LCA, namely: day 1 pupillary light reflex, corneal reflex, oculocephalic reflex, grimacing to pain, cough reflex, miosis and GCS eye and motor responses, we constructed a prognostic score called "Brainstem Responses Assessment Sedation score" (BRASS). Multiple logistic regression with a backward selection model was used with a p-value cut off at 0.15. Once variables of interest had been selected, two-way interactions between selected variables were then tested. Goodness-of-fit of the model was checked using le Cessie and van Houwelingen’s test [24]. Internal validation of the model, assessing robustness of the model selection procedure and bias correction of model calibration was performed by bootstrapping (200 bootstrap samples; S2 Fig; [25]). Once the final model selected, scores were attributed to the variables by roughly rounding the regression coefficients to the nearest integer (S2 Table; see also S2 Appendix for more details). Since the highest category of the model only contained one patient, the highest and the second highest categories of the model were combined. 28-day mortality was subsequently compared between the different score categories. External validation of the BRASS was performed using a subset of 46 deeply sedated patients from a previous study [14]. The discriminative ability of a model was assessed using the c-index (identical to the area under the receiver operating characteristics (ROC) curve [26]). No data for calculating the sample size for a latent class analysis or development of a prognostic score was available. We therefore included in the current study a number of patients similar to that of our previous study which assessed the prognostic value of the abolition of the cough reflex in deeply sedated ICU patients [14]. All tests were two-sided. Categorical variables were expressed as numbers (percentage), quantitative variables as median (first to third quartile, Q1 to Q_3_) or mean (standard deviation, SD). Analyses were performed using the R statistical software version 3.0.2 [27]. Latent class analyses were performed using the poLCA package [28].

## Results

### Description of the population

**Fig 1:**
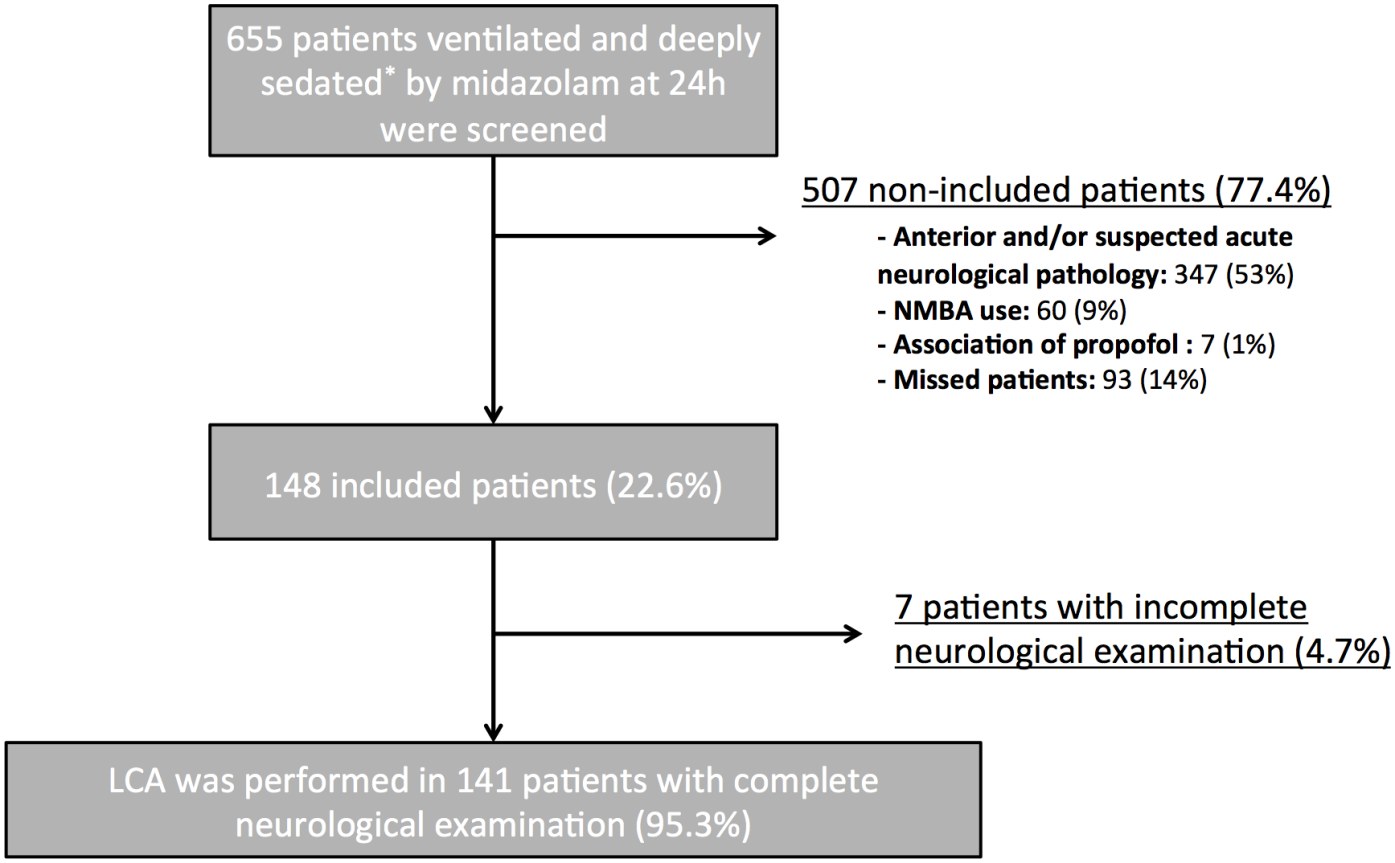
Flow chart. NMBA: neuromuscular-blocking agent; RASS: Richmond Assessment Sedation Scale.

Among 655 consecutive patients receiving deep sedation within 12 hours of admission, a total of 507 patients were excluded, mainly because of a preexisting and/or current acute neurological disease (n = 347, Fig 1). Overall, 148 patients were enrolled. At the time of inclusion, the RASS was -5 in 97 (66%) patients and -4 in 51 (44%) patients (Table 1). Median [interquartile range Q_1_ to Q_3_] duration of midazolam administration was 4 [3 to 7] days and awakening followed after 1 [0 to 2] days (S1 Fig). The most consistently found reflex in our cohort was the corneal reflex. By opposition, the OCR was the most frequently abolished reflex (Table 2). Demographic data, severity of critical illness, cumulative doses of midazolam and sufentanil, RASS and neurological examination did not differ significantly between the cohorts of each period.

**Table 1:** Patient characteristics.

Characteristic	*N = 148*
Age, mean (SD) ys	67.4 (15.4)
Gender, female, no. (%)	58 (39)
SAPS-II, median (Q_1_ to Q_3_)	56 (41 to 72)
Diagnosis at admission to the ICU, no. (%) ^a^	
Septicshock	55 (39)
ARDS	31 (22)
Severe sepsis	27 (19)
Hemorrhagic shock	6 (4)
Cardiogenic shock	2 (1)
Acute exacerbation of COPD	5 (4)
Other	16 (11)
Initiation of mechanical ventilation, no. (%) ^b^	
Acute respiratory failure	91 (65)
Shock	28 (20)
Surgery	12 (9)
Other	10 (7)
Initiation of sedation, no. (%) ^a^	
Synchrony with the ventilator	129 (91)
Agitation	1 (1)
Analgesia	12 (8)
SOFA-renal at day 1, median (Q_1_ to Q_3_)	1 (0 to 3)
SOFA-liver at day 1, median (Q_1_ to Q_3_)	0 (0 to 1)
SOFA-total at day 1, median (Q_1_ to Q_3_)	12 (8 to 15)
Surgical admission, no. (%)	33 (22)
Sepsis, no. (%)^c^	112 (76)
Pre-Deleric Score, median (Q_1_ to Q_3_) ^d^	0.64 (0.54 to 0.73)
Pre-sedation GCS, median (Q_1_ to Q_3_) ^d^	14 (11 to 15)
**Outcome**	
Duration of mechanical ventilation, median (Q_1_ to Q_3_) days	8 (5 to 16)
Duration of midazolam administration, median (Q_1_ to Q_3_) days	4 (3 to 7)
Time to awakening, median (Q_1_ to Q_3_) days ^e^	1 (0 to 2)
Occurrence of delirium, no. (%) ^f^	62 (56)
Occurrence of delayed awakening, no. (%) ^g^	41 (38)
Length of stay in the ICU, median (Q_1_ to Q_3_) days	14 (8 to 27)
Death at day 28, no. (%)	44 (30)
Death in the ICU, no. (%)	55 (37)

^a^ Data missing for six patients;

^b^ Data missing for seven patients.

^c^ ICU admission related to a proven or highly suspected bacterial infection.

^d^ PREdicition of DELirium in ICu patients. The Pre-Deleric predicts the subsequent occurrence of delirium at the time of ICU admission [17,18].

^e^ Interval of time between midazolam discontinuation and awakening. Awakening was defined by eye opening and visual contact >10 sec (RASS ≥ -1). Awakening could occur before discontinuation of sedation.

^f^ Delirium was defined using the CAM-ICU [21].

^g^ Delayed awakening was defined by RASS<-1 within 3 days of discontinuation of sedation. ICU: intensive care unit; SAPS-II: Simplified Acute Physiology Score II; ARDS: Acute Respiratory Distress Syndrome; COPD: Chronic Obstructive Pulmonary Disease; SOFA: Sequential Organ Failure Assessment; RASS: Richmond Assessment Sedation Scale; Data are presented as median (interquartile range Q_1_ to Q_3_) or number (percent).

**Table 2:** Neurological assessment at the time of inclusion.

Sedation (cumulative dose)	*N = 148* *
Midazolam, median (Q_1_ to Q_3_) g/kg**	1.3 (0.8 to 2.0)
Sufentanil, median (Q_1_ to Q_3_) μg/kg**	2.3 (1.4 to 4.3)
RASS -5	97 (66)
**Neurologic response**	
GCS Motor, median (Q_1_ to Q_3_)	1 (1 to 2)
GCS Ocular, median (Q_1_ to Q_3_)	1 (1 to 1)
FOUR-score	4 (3 to 5)
Eye response	0 (0 to 0)
Motor response	0 (0 to 1)
Brainstem reflexes	4 (2 to 4)
Respiration	0 (0 to 1)
Brainstem reflexes (response resent)	
Pupillary light reflex, no. (%)	113 (77)
Corneal reflex, no. (%)	127 (86)
Oculocephalic reflex, no. (%)	62 (43)
Grimacing to pain, no. (%)	79 (54)
Cough reflex, no. (%)	107 (73)
Miosis, no. (%)	95 (65)

* Data was incomplete in one (for Midazolam, Sufentanil, RASS, Pupillary light reflex, Cough reflex and Miosis) two (for FOUR-score, Grimacing to pain) and three (for Oculocephalic reflex) patients.

**Cumulative dose from onset of sedation to neurological examination (i.e. inclusion).

GCS: Glasgow Coma Scale; Data are presented as median (interquartile range Q_1_ to Q_3_) or number (percent).

### Classification of patients by latent class analysis (LCA)

**Fig 2:**
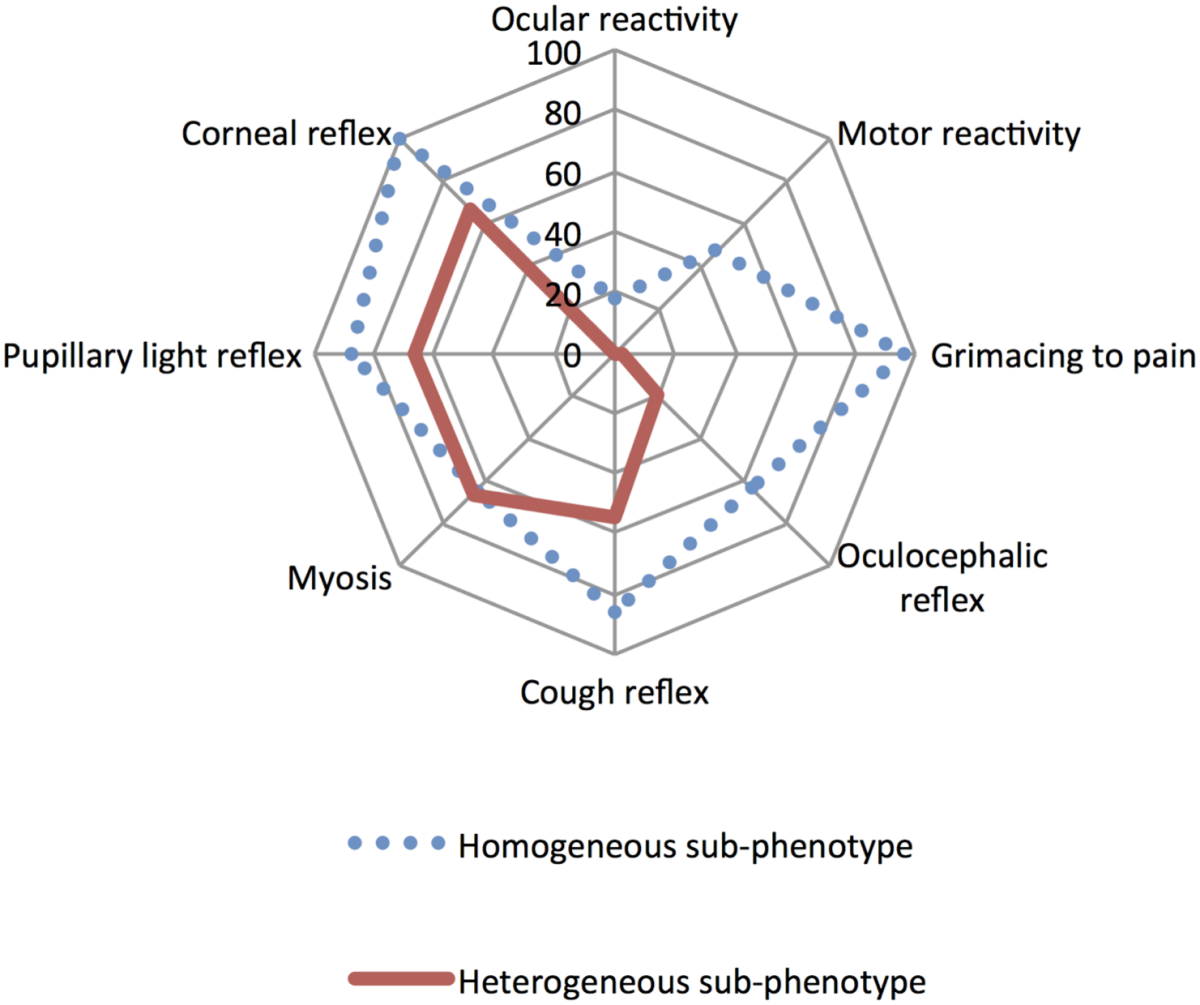
Representation of homogeneous and heterogeneous profiles. The percentage of abolition of each tested neurological responses in homogeneous and heterogeneous profiles are depicted. The heterogeneous profile is characterized by a greater and more heterogeneous abolition of neurological responses.

Brainstem reflex assessment was incomplete in seven patients. We performed LCA in 141 (95%) patients, which took exclusively into account neurological responses (Table 3). Two sub-phenotypes were identified, in 77 (54.6%) and 64 (45.4%) patients and were respectively called homogeneous and heterogeneous brainstem reactivity profiles. In comparison to the homogeneous profile, heterogeneous profile was characterized by less ocular and motor reactivity to nociceptive stimulation. The heterogeneous sub-phenotype was also associated with a decrease in proportion of preserved pupillary light, corneal and cough reflexes as well as the abolition of a large proportion of both grimace in response to pain and OCR (Fig 2). Neither the period nor the center of inclusion accounted for observed differences between homogeneous or heterogeneous sub-phenotypes. Patients with a heterogeneous profile were mainly admitted for a medical cause and presented with higher severity scores (Table 3). Cumulative administered doses of midazolam and sufentanil at the time of inclusion were comparable between the two profiles, however the proportion of patients with a RASS of -5 was significantly greater in the heterogeneous profile (Table 3). The median duration of sedation, the median time to awakening and the proportion of non-sedated patients on day 4, did not statistically differ between the two sub-phenotypes (Table 3).

**Table 3:** Latent class description and outcomes.

Sub-phenotypes	Homogeneous	Heterogeneous	p
Number (%) of patients	77 (54.6)	64 (45.4)	
**Neurologic responses** (parameters used in LCA)			
GCS Motor, median (Q_1_ to Q_3_)	1 (1 to 4)	1 (1 to 1)	<0.0001
No. (%) ≥2>+2	36 (47)	0 (0)	<0.0001
GCS Ocular, median (Q_1_ to Q_3_)	1 (1 to 1)	1 (1 to 1)	0.0004
No. (%) ≥2	14 (18)	0 (0)	0.0001
Brainstem reflexes (response present)			
Pupillary light reflex, no. (%)	67 (87)	42 (66)	0.0004
Corneal reflex, no. (%)	77 (100)	43 (67)	<0.0001
Oculocephalic reflex, no. (%)	49 (64)	13 (20)	<0.0001
Grimacing to pain, no. (%)	74 (96)	2 (3)	<0.0001
Cough reflex, no. (%)	66 (86)	35 (55)	<0.0001
Miosis, no. (%)	50 (65)	42 (66)	>0.99
FOUR-score	5 (4 to 7)	3 (2 to 4)	<0.0001
**Patient’s characteristics**			
Age, mean (SD) ys	65.1 (15.9)	69.3 (14.7)	0.14
Gender, female, no. (%)	34 (44.2)	23 (35.9)	0.39
Surgical admission, no. (%)	23 (30)	9 (14)	0.028
Sepsis, no. (%) ^a^	58 (75)	50 (78)	0.84
SAPS-II, median (Q_1_ to Q_3_)	51 (36 to 65)	58 (47 to 77)	0.013
SOFA-renal at day 1, median (Q_1_ to Q_3_)	0 (0 to 1)	2 (0 to 3)	<0.0001
SOFA-liver at day 1, median (Q_1_ to Q_3_)	0 (0 to 1)	0 (0 to 2)	0.10
SOFA-total at day 1, median (Q_1_ to Q_3_)	10 (7 to 13)	14 (11 to 16)	<0.0001
Pre-Deleric Score, median (Q_1_ to Q_3_) ^b^	0.59 (0.46 to 0.7)	0.67 (0.59 to 0.76)	0.001
Pre-sedation GCS, median (Q_1_ to Q_3_) ^b^	14 (11 to 15)	14 (11 to 15)	0.25
**Sedation (cumulative dose)**			
Midazolam, median (Q_1_ to Q_3_) mg/kg ^c^	1.3 (0.8 to 2.0)	1.3 (0.7 to 1.9)	0.84
Sufentanil, median (Q_1_ to Q_3_) μg/kg^c^	2.4 (1.3 to 4.4)	2.2 (1.5 to 4.4)	0.63
RASS -5	40 (52)	51 (80)	0.0007
Increased P14-N20, no. (%) ^d^	5/14 (35.7)	6/14 (42.8)	>0.99
Increased III-V, no. (%) ^d^	0/14 (0)	3/14 (21.4)	0.22
Increased P14-N20 and/or III-V, no. (%) ^d^	5/14 (35.7)	8/14 (57.1)	0.69
**Outcome**			
Duration of mechanical ventilation, median (Q_1_ to Q_3_) days	9 (6 to 17)	8 (5 to 15)	0.29
Duration of midazolam administration, median (Q_1_ to Q_3_) days	4 (3 to 8)	4 (3 to 6)	0.79
Time to awakening, median (Q_1_ to Q_3_) days ^e^	1 (0 to 2)	1 (1 to 2)	0.10
Patient free of sedation at day 4, no (%) ^h^	52 (68.4)	31 (68.9)	0.99
Occurrence of delirium, no. (%) ^f^	34 (49)	25 (68)	0.10
Occurrence of delayed awakening, no. (%) ^g^	14 (24)	26 (59)	0.0004
Length of stay in the ICU, median (Q_1_ to Q_3_) days	15 (10 to 29)	11 (6 to 21)	0.02
Death at day 28, no. (%)	9 (12)	33 (52)	<0.0001^d^
Death in the ICU, no. (%)	15 (19)	38 (59)	<0.0001

Latent class analysis (LCA) was performed on neurological responses assessed at day-1 only (8 variables in the bold rectangle).

^a^ ICU admission related to a proven or highly suspected bacterial infection.

^b^ PREdicition of DELirium in ICu patients. The Pre-Deleric predicts the subsequent occurrence of delirium at the time of ICU admission [17,18].

^c^ Cumulative dose from onset of sedation and neurological examination (i.e. inclusion)

^d^ Somatosensory evoked potentials enabled to assess the P14-N20 interlatency, which reflects the medullo-cortical conduction time; Auditory evoked potentials allowed to assess the III-V interlatency, which reflect the ponto-mesencephalic conduction time. The neurophysiological tests were performed in 28 (19%) patients.

^e^ Awakening was defined by eye opening and visual contact >10 sec (RASS ≥ -1). Awakening could occur before discontinuation of sedation

^f^ Delirium was defined using the CAM-ICU [21].

^g^ Delayed awakening was defined by RASS<-1 within 3 days following the discontinuation of sedation.

^h^ Proportion of patients free of sedation among patients alive at day 4.

ICU: intensive care unit; SAPS-II: Simplified Acute Physiology Score II; SOFA: Sequential Organ Failure Assessment; GCS: Glasgow Coma Scale; FOUR: Full Outline Of Unresponsiveness; RASS: Richmond Assessment Sedation Scale.

Brain imaging was performed in 24 (16%) patients, including 13 MRI and 25 CT scans, and was considered normal in 12/24 (50%) cases. No brainstem lesion was observed (non-specific periventricular white matter lesions (8/24, 33.3%) and brain atrophy (6/24, 25%) were the most frequent findings; ischemic stroke was observed in 2 patients). Evoked potentials, performed in 28 (19%) patients, displayed increased medullo-cortical and/or ponto-mesencephalic conduction times in 13 patients (46%; Table 3).

### Relationship between clinical profiles and outcomes

Forty-four (30%) patients died by day 28, mainly because of multiple organ failure (n = 34, 60%) or refractory hypotension (n = 9, 16%). Limitations of active treatments were decided for 14 (9%) patients, but were never based on the results of neurological examination on day 1. Twenty-eight-day mortality was significantly higher in the heterogeneous profile than in the homogeneous profile (52% *versus* 12%, respectively, p<0.0001, Table 3). The heterogeneous sub-phenotype was associated with increased 28-day mortality, after adjustment on SAPS-II (Odds Ratio [95% confidence interval] = 6.93 [2.88–16.7], p <0.0001), SAPS-II and RASS (6.44 [2.63–15.8], p <0.0001), SOFA (5.13 [2.08–12.6], p = 0.0004) or SOFA and RASS (5.02 [2.01–12.5], p = 0.0005) (primary objective; Table 4). When restricting the analysis to patients with RASS–5, the heterogeneous profile remained associated with 28-day mortality, after adjustment on SAPS-II or SOFA (Table 4).

**Table 4:** Association between heterogeneous sub-phenotype and 28-day mortality.

	All patients (N = 141)		RASS = -5 (N = 91)	
	OR (95% CI)	p	OR (95% CI)	p
**Unadjusted Latent Class Analysis (LCA)**				
Heterogeneous sub-phenotype	8.04 (3.44 to 18.8)	<0.0001	6.37 (2.28 to 17.8)	0.0004
**LCA adjusted to the SAPS II**				
SAPS II	1.03 (1.01 to 1.06)	0.003	1.02 (1.00 to 1.05)	0.050
Heterogeneous sub-phenotype	6.93 (2.88 to 16.7)	<0.0001	5.82 (2.04 to 16.6)	0.001
**LCA adjusted to SAPS-II and RASS**				
SAPS II	1.03 (1.01 to 1.06)	0.004		
RASS -5	1.41 (0.54 to 3.72)	0.48		
Heterogeneous sub-phenotype	6.44 (2.63 to 15.8)	< 0.0001		
**LCA adjusted to SOFA**				
SOFA	1.21 (1.07 to 1.38)	0.003	1.14 (1.00 to 1.31)	0.055
Heterogeneous sub-phenotype	5.13 (2.08 to 12.6)	0.0004	4.69 (1.60 to 13.7)	0.005
**LCA adjusted to SOFA and RASS**				
SOFA	1.21 (1.06 to 1.38)	0.004		
RASS—5	1.13 (0.42 to 3.03)	0.80		
Heterogeneous sub-phenotype	5.02 (2.01 to 12.5)	0.0005		

LCA: Latent Class Analysis; SAPS-II: Simplified Acute Physiology Score II; SOFA: Sequential Organ Failure Assessment; RASS: Richmond Assessment Sedation Scale.

Delirium occurred in 62 (56%) and delayed awakening in 41 (38%) patients (Table 1). The prevalence of delirium did not significantly differ between homogeneous and heterogeneous profiles (49% *vs*. 68%, p = 0.10), while delayed awakening was significantly less frequent in the homogeneous profile (24% *vs*. 59%, p = 0.0004; Table 3). Overall, altered mental status following sedation discontinuation was significantly more frequent in the heterogeneous profile (64% *vs*. 89%, p = 0.002).

### Development and validation of the Brainstem Responses Assessment Sedation Score (BRASS)

**Fig 3:**
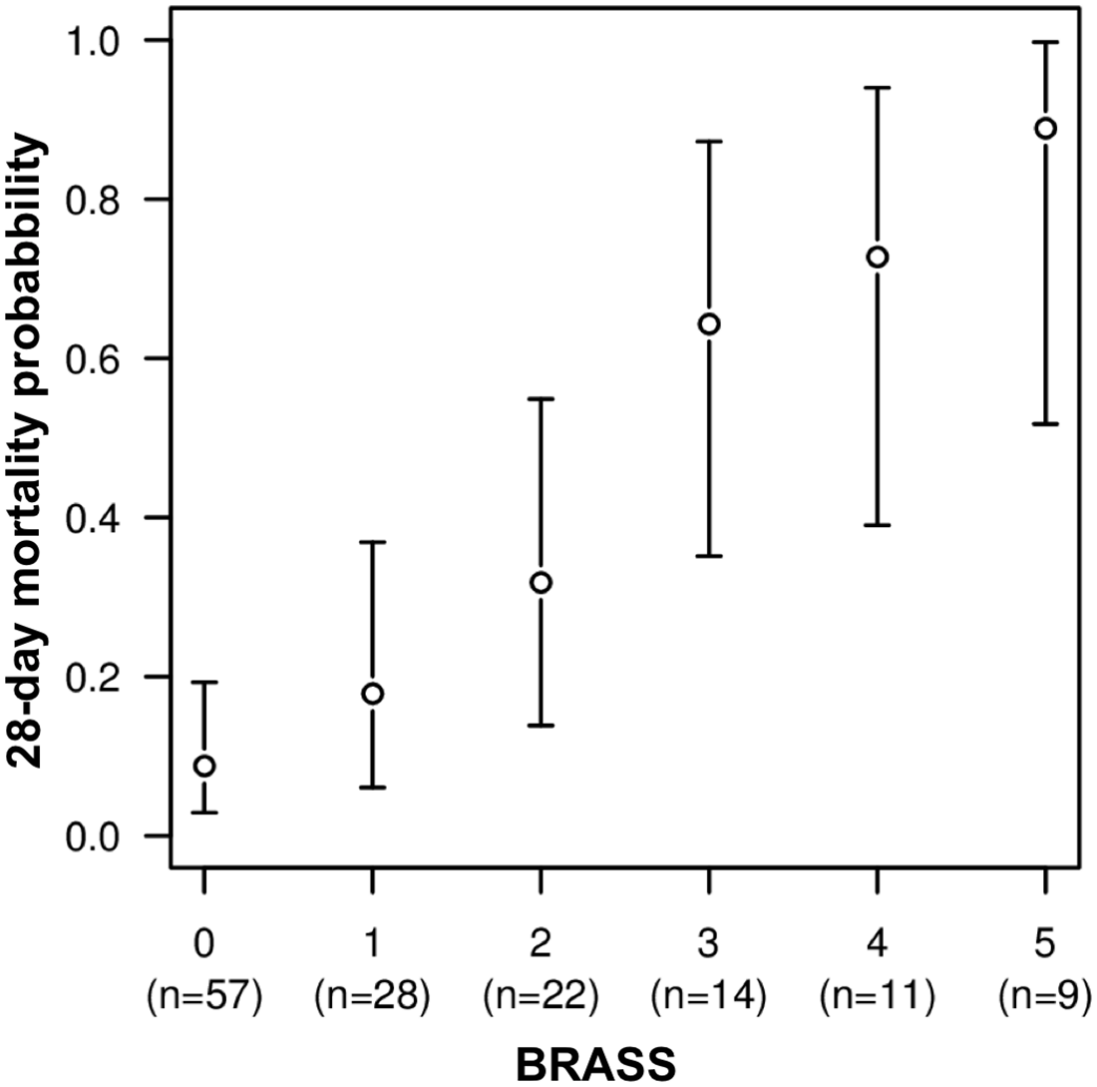
28-day mortality probability according to the BRASS. Mortality probability expressed in mean [95%CI], BRASS: Brainstem Responses Assessment Sedation Score.

After rounding of logistic regression coefficients, absence of pupillary light reflex, cough reflex and the combined absence of grimace and OCR were each attributed a score of 1. Absent corneal reflex was attributed a score of 2 and absent grimace in the presence of OCR was attributed a score of 3. The resulting sum theoretically ranged from 0 to 7, but since only one patient in our cohort obtained a score over 5, we set a maximum value for the BRASS at 5 (Fig 3). Both the BRASS and FOUR-score reliably predicted 28-day mortality, with a higher likelihood ratio for the BRASS than the FOUR-score (49.5 *vs*. 37.1) and better discrimination, with c-indexes [95%CI] of 0.82 [0.74–0.90] for the BRASS *vs*. 0.76 [0.67–0.84] for the FOUR-score, respectively, though the difference in c-index did not reach statistical significance (p = 0.10; S3 Table). The predictive value of the BRASS and FOUR-score was validated using the historical cohort of 46 sedated patients with RASS<-3, with c-indexes of 0.69 [0.54–0.84] for the BRASS and 0.65 [0.49–0.80] for the FOUR-score, respectively (NS). This historical cohort was comparable to the other cohorts of the present study regarding the SAPS-II (52 [40–63]), SOFA (11 [10–15]), RASS (-5 [-5 to -5]) and 28-day mortality rate (n = 16 [34%]) but not for age (67.0 ± 15.4 vs 59.7 ± 15.6, p = 0.004). Motor reactivity (1 [1 to 2] *versus* 1 [1 to 1], p = 0.015) and cough reflex (72% versus 51%, p = 0.03) were more frequently abolished in the historical cohort.

The probability of 28-day mortality was improved in a model combining the SAPS-II and BRASS compared to the SAPS-II alone (Net Reclassification Improvement (NRIc) 0.984, SE 0.200, p<0.0001; S4 Table). Similarly, the probability of 28-day mortality was improved in a model combining the SOFA and BRASS compared to the SOFA alone (NRIc 1.004, SE 0.184, p<0.0001).

Brainstem reflexes were also assessed on day 4 in 42 (61%) patients among the 69 (47%) who had still a RASS < -3. All brainstem reflexes were present in more than 80% of patients, except for the grimace and OCR which were detected in about 60% of the patients (S5 Table). Finally the BRASS calculated at day 4 was significantly greater in non-survivors than survivors (1 [0 to 2] *versus* 0 [0 to 0.25], p = 0.027).

## Discussion

The present study indicates that, in non-brain injured deeply sedated mechanically ventilated patients, the motor and ocular reactivity to nociceptive stimuli combined with brainstem responses can be classified into two separate clinical sub-phenotypes (i.e. homogeneous and heterogeneous profiles). The former is characterized by a decreased reactivity to nociceptive stimuli associated with homogeneous brainstem responses. The latter is characterized by a loss of reactivity to GCS nociceptive stimuli and a heterogeneous abolition of brainstem responses, involving the grimace, OCR and to a lesser extent the cough reflex. The heterogeneous sub-phenotype is a predictor of 28-day mortality, after adjustment on severity scores (i.e. SAPS-II and SOFA) and RASS. This prediction of mortality is also found within the subgroup of deepest sedated patients. In order to ensure that our phenotypic approach was clinically relevant, we developed a clinical score (BRASS) in aiming to help physicians to assess brainstem reactivity in a given deeply sedated patient. We found that the BRASS and the FOUR-score had a relatively similar predictive value for 28-day mortality.

Overall, our findings help enhance the neurological assessment and prognostication of deeply sedated non-brain injured mechanically ventilated critically ill patients. Indeed, the neurological items incorporated in commonly used severity scores (SAPS-II and SOFA) are those of the GCS, which does not include brainstem reflexes. Similarly, the assessment of brainstem reflexes is not integrated into the RASS, which only assesses reactivity to verbal and non-nociceptive physical stimulation. From a neurological, our approach is similar to that proposed in comatose patients, where the assessment of brainstem reflexes completes the GCS. Here, the assessment of brainstem responses completes the RASS in deeply sedated critically ill patients. Therefore, our findings are likely to contribute to improving the care of deeply sedated patients, since the assessment of brainstem reflexes can easily be performed by any ICU physician. Our findings may also contribute to some extent to account for the relationship between deep sedation and increased mortality reported by Shehabi and colleagues, inasmuch as brainstem reflexes were not assessed in their study [6].

We hypothesized that the heterogeneous sub-phenotype may reflect a dysfunction of the brainstem, accounting for mortality and delirium/delayed awakening since the brainstem controls both vital functions and arousal. Evidence from our study and from previous studies may suggest a dysfunction of the brainstem in critically ill patients. Critical illness is associated with a degree of autonomic dysfunction such as impaired heart rate variability, which is associated with increased mortality [29]. The increased brainstem conduction time that we observed is consistent with this hypothesis, although evoked potentials were only assessed in a restricted sample of subjects (Table 3).

The respective role of the general sickness and of deep sedation in the genesis of both sub-phenotypes is unclear. Firstly, the heterogeneous profile does not match any previously described syndrome and may not be explained by any single focal injury of the brainstem. Indeed, abolition of the OCR, grimace and cough reflex indicates involvement of the midbrain, pons and medulla. However, such an extensive brainstem injury is ruled out by the absence of an upper motor neuron syndrome and no visible sub-tentorial abnormalities on brain imaging (while acknowledging that the CT scan is not the most reliable method for investigating the brainstem). The heterogeneous sub-phenotype is therefore likely to rely on a functional rather than a structural origin; it can be therefore speculated that some neuro-anatomical centers are more sensitive to deep sedation, critical illness or both. The arguments in favor of a role of general sickness in generating the heterogeneous profile are that the infusion rate of midazolam did not differ between the two sub-phenotypes; that severity scores were greater in the heterogeneous profile and the fact that brainstem conduction times were increased, and these measurements are not modified by midazolam [30,31]. Additional arguments stem from previous studies. A systemic inflammatory response may trigger neuro-inflammation, as circulating mediators can reach the brainstem through the *area postrema* [32]. Thus, neuro-inflammation and neuronal apoptosis have been evidenced within the brainstem in fatal cases of septic shock [33–35]. These processes are infra-radiological, explaining why brain imaging does not reliably succeed in detecting insults to the brainstem. Deep sedation may also play a role in generating the heterogeneous profile; indeed most patients were conscious prior to the initiation of sedation and brainstem reflexes generally reappeared following cessation of sedation (S5 Table). It may even be speculated that the heterogeneous profile is in fact an up-to-now unidentified level of deep sedation, not included in the RASS, reflecting over-sedation. Such over-sedation may be related to impaired drug clearance. Indeed renal and hepatic failures were observed more frequently in the heterogeneous profile. A trial assessing plasma levels of midazolam and sufentanil might help confirm this hypothesis. Given these arguments and the fact that our study is strictly observational, we definitely cannot delineate the respective roles of the initial sickness versus deep sedation in generating the heterogeneous profile. Further clinical and experimental studies on the subject are needed (a larger multicenter prospective study is ongoing; ClinicalTrials.gov number: NCT02395861).

### Limitations of the study

Our study has limitations. First, one may question the generalizability of our findings as they were obtained in a selected population of deeply sedated mechanically ventilated critically ill patients. Also, the BRASS was validated with a c-index of 0.69 in a restricted historical cohort, but exhibited c-stats parameters not different from the FOUR-scores.

Second, the purpose of our study was in no way to promote the use of midazolam or of early deep sedation, which may have deleterious effects [4,6,9]. The indications for deep sedation in our patients were based on particularly severe situations where patients were obviously highly uncomfortable or experiencing agitation, ventilator asynchrony or severely impaired gas exchange. Recent studies indicate that ICU physicians use deep sedation in more than 30% of sedated patients and that midazolam remains one the most commonly used sedative agents together with propofol, and in combination with opioids [4–7,36]. Management of sedation, and severity of critical illness in our study are comparable to those reported elsewhere [1,4,37]. This suggests that our population may be representative of deeply sedated ICU patients. It would be of interest to assess neurological profiles in patients sedated by propofol, which shares common molecular mechanisms with midazolam by acting at the GABA-A receptor-coupled chloride channel.

Third, patients’ characteristics, neurological responses, sedation regimens and outcomes did not differ between centers, observers and study periods. This suggests that our results may be extrapolated to other ICUs and other physicians.

Fourth, the discrepancy in the prevalence of abolished cough reflex between the historical and the development cohorts could account for the relative weakness of BRASS validation. This discrepancy might be related to the small size of the validation cohort but also to the fact that patients of the historical group were less reactive to nociceptive stimulation [38]. All in all, the BRASS remains efficient, as a c-index of 0.69 [0.54 to 0.84] denotes fair discrimination, supporting the generalizability of our findings. Interestingly, we also found that BRASS at day 4 remained associated with mortality.

Fifth, LCA is an exploratory rather than a confirmatory procedure. LCA is associated with a risk of reification, where identified latent classes are considered to be the actual types of individuals in the population. Although it might be considered relatively small, the sample size of our cohort was large enough to compute LCA [39]. It has to be noted that characteristics of the sub-populations and management of sedation did not differ between centers or study periods.

## Conclusion

We report, in approximately 50% of non-brain injured mechanically ventilated deeply sedated critically ill patients, a particular neurological pattern within 24 hours of sedation, characterized by a loss of reactivity to nociceptive stimuli and a heterogeneous abolition of brainstem reflexes. When adjusted to severity scores, depth of sedation, this sub-phenotype was a robust predictor of increased 28-day mortality, Our findings may suggest the existence of a brainstem dysfunction that can be clinically detected using the BRASS or the FOUR-score.

## Supporting information

S1 Fig Proportion of sedated (a) and awake (b) patients over the first 28-day following admission to the ICU. In a), patients were classified into four categories: 1) alive under sedation, 2) dead under sedation, 3) dead following discontinuation of sedation, 4) alive following discontinuation of sedation. In b), patients were classified into four categories: 1) alive not awake, 2) dead not awake, 3) dead after awakening, 4) alive after awakening. Awakening was defined by eye opening and visual contact >10 sec (RASS ≥ -1).
(TIFF)

S2 Fig Calibration plot for the model with neurologic responses*. The solid and dotted lines plot the actual versus model-predicted 28-day mortality. The dashed line represents perfect calibration. * Neurological responses included in the BRASS: pupillary light reflex, corneal reflex, grimace in response to pain, oculocephalic and cough reflexes.
(TIFF)

S1 Table Indices for latent class models ranging from 1 to 5 classes. The bootstrap likelihood ratio test (BLRT) yielded p<0.001 for a model with 2 vs 1 class, p = 0.085 for 3 vs 2 classes and p = 0.033 for 4 vs 3 classes. AIC: Akaike Information Criterion, BIC: Bayesian Information Criterion.
(DOCX)

S2 Table Construction of the Brainstem Responses Assessment Sedation Score (BRASS). The score range from 0 to 7; OCR: oculephalic reflex.
(DOCX)

S3 Table Association of the BRASS and FOUR score with 28-day mortality. The p-values compare the c-index of the models to the model with SAPS-II only. SAPS-II: Simplified Acute Physiology Score II; BRASS: Brainstem Responses Assessment Sedation Score; FOUR: Full Outline Of Unresponsiveness.
(DOCX)

S4 Table Statistical indexes of the final models. AIC and BIC for models with SAPS-II, BRASS and both, as well as the NRIc and IDI for a model with the BRASS compared to a model with SAPS-II alone. Results show a significant improvement of BRASS as compared to SAPS-II. AIC: Akaike Information Criterion, BIC: Bayesian Information Criterion, NRIc: Net Reclassification Improvement, IDI: Integrated Discrimination Improvement.
(DOCX)

S5 Table Brainstem reflexes at day 4 among patients deeply sedated (RASS < -3). Neurological examination was available in 42 out of 69 patients who were alive and had a RASS < - 3 at day 4. Neurological examination was not systematically performed after day 1 in patients with RASS remained below -3. It has to be reminded that brainstem reflexes were never assessed in patients with RASS ≥– 3. All brainstem reflexes were more frequently present on day 4 than on day 1 but also in patients without than with midazolam, except for the cough reflex. Discontinuation of sedation does not imply that there was no persisting effect of midazolam. Mid+: patient receiving continuous midazolam infusion.
(DOCX)

S1 Appendix Brainstem responses assessment in ICU Form. (PDF)

S2 Appendix Details regarding the calibration of the BRASS. (DOCX)
