# Supplementary figures and images for: Brainstem response patterns in deeply-sedated critically-ill patients predict 28-day mortality
Source: PLoS One. 2017 Apr 25;12(4):e0176012. doi: 10.1371/journal.pone.0176012 (PMC5404790; doi:10.1371/journal.pone.0176012)

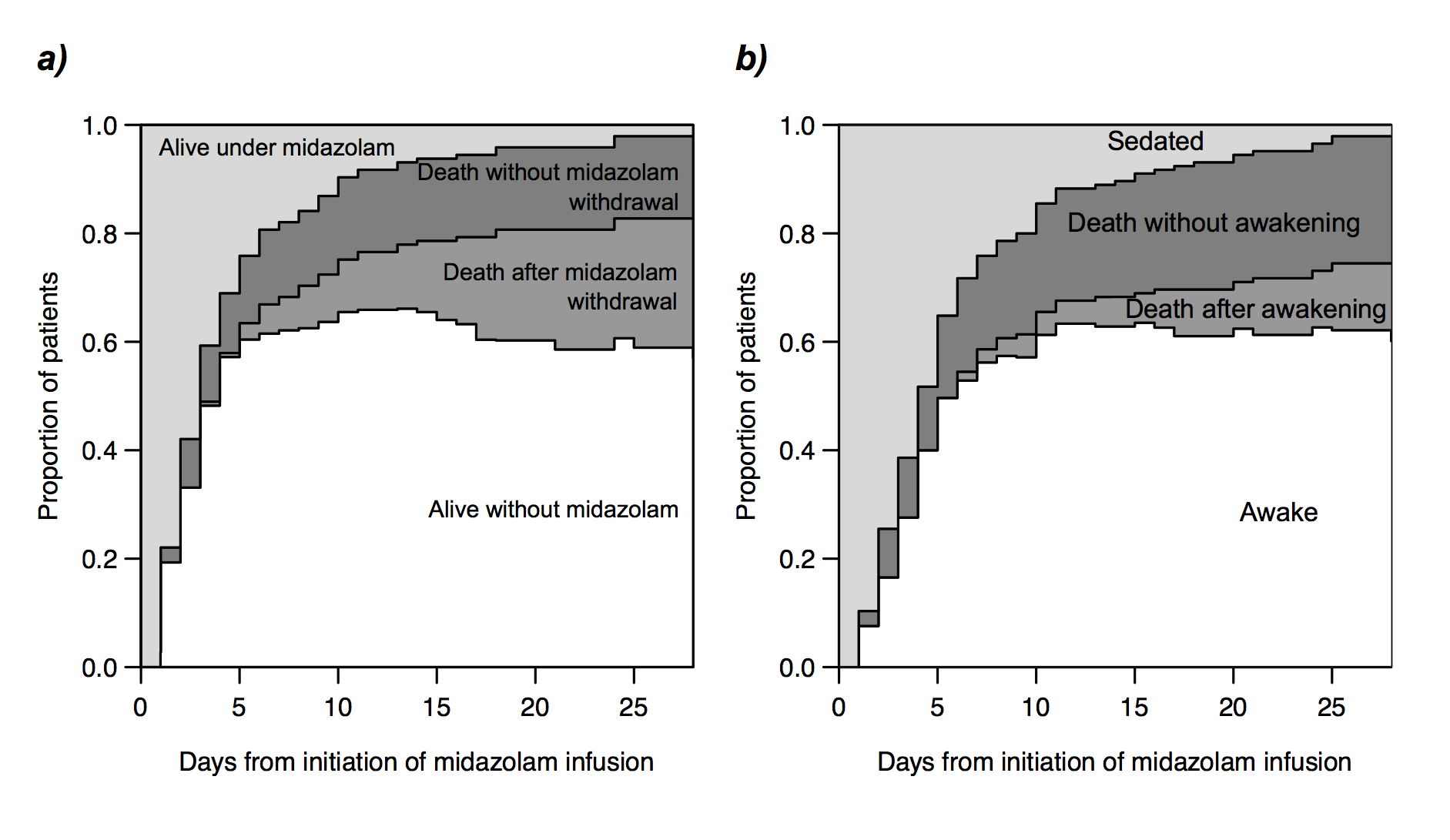

Supplement: S1 Fig — In a), patients were classified into four categories: 1) alive under sedation, 2) dead under sedation, 3) dead following discontinuation of sedation, 4) alive following discontinuation of sedation. In b), patients were classified into four categories: 1) alive not awake, 2) dead not awake, 3) dead after awakening, 4) alive after awakening. Awakening was defined by eye opening and visual contact >10 sec (RASS ≥ -1). (TIFF) [file pone.0176012.s001.tiff]

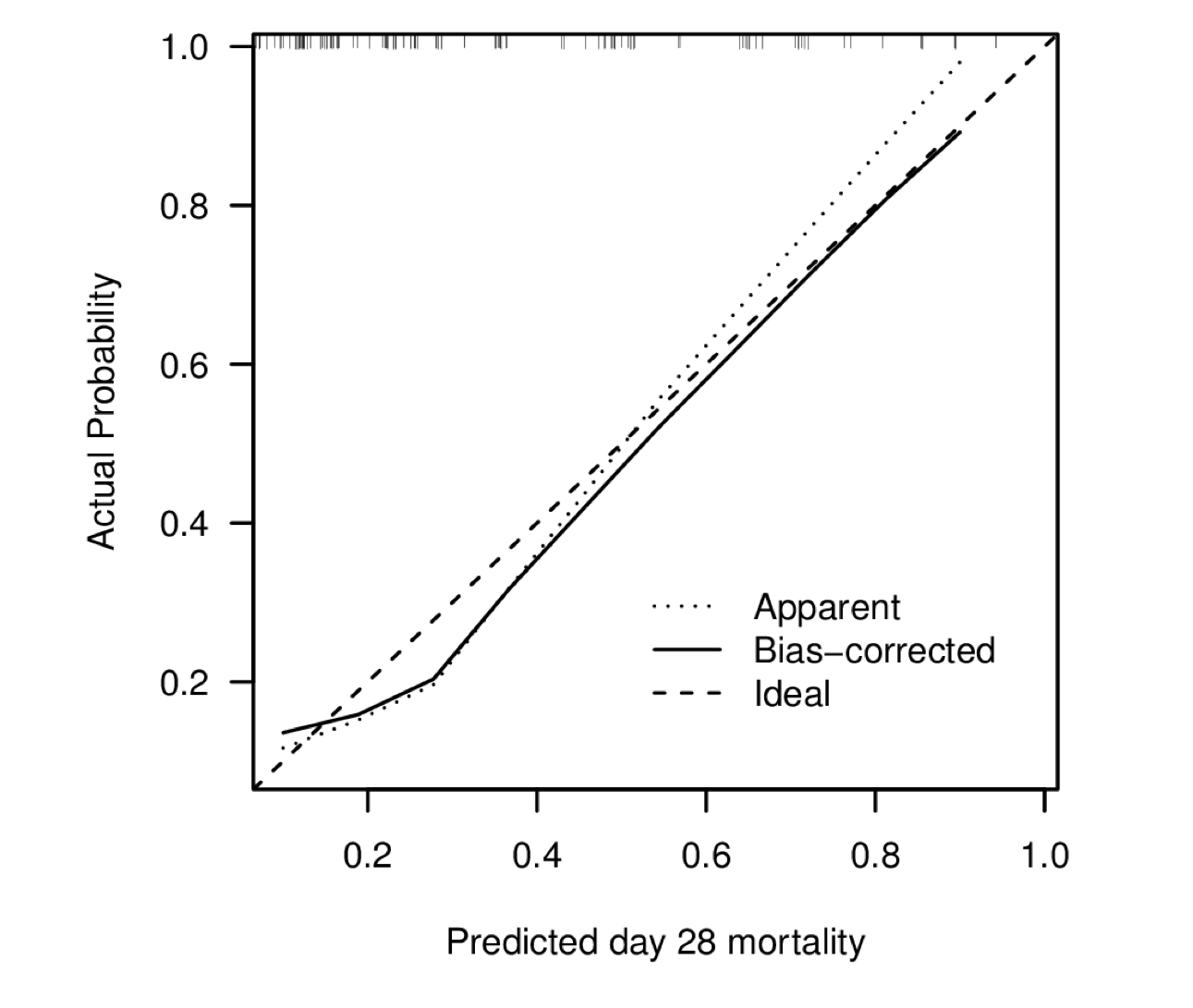

Supplement: S2 Fig — The solid and dotted lines plot the actual versus model-predicted 28-day mortality. The dashed line represents perfect calibration. * Neurological responses included in the BRASS: pupillary light reflex, corneal reflex, grimace in response to pain, oculocephalic and cough reflexes. (TIFF) [file pone.0176012.s002.tiff]
