# Supplementary material for: Brainstem response patterns in deeply-sedated critically-ill patients predict 28-day mortality
Source: PLoS One. 2017 Apr 25;12(4):e0176012. doi: 10.1371/journal.pone.0176012 (PMC5404790; doi:10.1371/journal.pone.0176012)
Supplement: S1 Table — The bootstrap likelihood ratio test (BLRT) yielded p<0.001 for a model with 2 vs 1 class, p = 0.085 for 3 vs 2 classes and p = 0.033 for 4 vs 3 classes. AIC: Akaike Information Criterion, BIC: Bayesian Information Criterion. (DOCX) [file pone.0176012.s003.docx]

|  | **Number of latent classes** | | | | |
| --- | --- | --- | --- | --- | --- |
|  | One | Two | Three | Four | Five |
| **Pearson Chi²** | 446.8 | 144.1 | 116.6 | 84.2 | 67.6 |
| **Log-likelihood Chi² (G^2^)** | 239.8 | 109.3 | 90.6 | 71.1 | 62.4 |
| **Degrees of freedom (df)** | 133 | 124 | 115 | 106 | 97 |
| **Log-likelihood** | -629.7 | -564.5 | -555.2 | -545.4 | -541.1 |
| **No. parameters** | 8 | 17 | 26 | 35 | 44 |
| **AIC** | 1275.5 | 1163.0 | 1162.3 | 1160.9 | 1170.2 |
| **BIC** | 1299.1 | 1213.2 | 1239.0 | 1264.1 | 1299.9 |

**S1 Table. Indices for latent class models ranging from 1 to 5 classes.**

The bootstrap likelihood ratio test (BLRT) yielded p<0.001 for a model with 2 vs 1 class, p=0.085 for 3 vs 2 classes and p=0.033 for 4 vs 3 classes. AIC: Akaike Information Criterion, BIC: Bayesian Information Criterion.
