# Supplementary material for: Brainstem response patterns in deeply-sedated critically-ill patients predict 28-day mortality
Source: PLoS One. 2017 Apr 25;12(4):e0176012. doi: 10.1371/journal.pone.0176012 (PMC5404790; doi:10.1371/journal.pone.0176012)
Supplement: S2 Table — The score range from 0 to 7; OCR: oculephalic reflex. (DOCX) [file pone.0176012.s004.docx]

**S2 Table. Construction of the Brainstem Responses Assessment Sedation Score (BRASS).**

| **Variable** | **Adjusted OR (95% CI)** | **Score points** |
| --- | --- | --- |
| Absence of cough reflex | 2.15 (0.81 to 5.71) | 1 |
| Absence of pupillary light reflex | 3.31 (1.23 to 8.90) | 1 |
| Absence of corneal reflex | 4.07 (1.20 to 13.8) | 2 |
| Absence of grimacing to pain and absence of OCR | 2.52 (0.91 to 7.00) | 1 |
| Absence of grimacing to pain and presence of OCR | 24.1 (5.19 to 111.6) | 3 |

The score range from 0 to 7; OCR: oculephalic reflex.
