# Supplementary material for: Brainstem response patterns in deeply-sedated critically-ill patients predict 28-day mortality
Source: PLoS One. 2017 Apr 25;12(4):e0176012. doi: 10.1371/journal.pone.0176012 (PMC5404790; doi:10.1371/journal.pone.0176012)
Supplement: S3 Table — The p-values compare the c-index of the models to the model with SAPS-II only. SAPS-II: Simplified Acute Physiology Score II; BRASS: Brainstem Responses Assessment Sedation Score; FOUR: Full Outline Of Unresponsiveness. (DOCX) [file pone.0176012.s005.docx]

|  | **Fitting set** | | **Validation set** |
| --- | --- | --- | --- |
| **Model** | Likelihood ratio | c-index (95% CI) | c-index (95% CI) |
| SAPS-II | 15.60 | 0.69 (0.60 to 0.79) | 0.72 (0.55 to 0.89) |
| BRASS | 46.14 | 0.82 (0.74 to 0.90) | 0.69 (0.54 to 0.84) |
| BRASS and SAPS-II | 49.49 | 0.84 (0.77 to 0.92) | 0.74 (0.58 to 0.90) |
| FOUR score | 27.13 | 0.76 (0.67 to 0.84) | 0.65 (0.49 to 0.80) |
| FOUR score and SAPS-II | 37.13 | 0.81 (0.74 to 0.89) | 0.74 (0.58 to 0.90) |

**S4-Table. Association of the BRASS and FOUR score with 28-day mortality.**

The p-values compare the c-index of the models to the model with SAPS-II only. SAPS-II: Simplified Acute Physiology Score II; BRASS: Brainstem Responses Assessment Sedation Score; FOUR: Full Outline Of Unresponsiveness.
