# Supplementary material for: Brainstem response patterns in deeply-sedated critically-ill patients predict 28-day mortality
Source: PLoS One. 2017 Apr 25;12(4):e0176012. doi: 10.1371/journal.pone.0176012 (PMC5404790; doi:10.1371/journal.pone.0176012)
Supplement: S4 Table — AIC and BIC for models with SAPS-II, BRASS and both, as well as the NRIc and IDI for a model with the BRASS compared to a model with SAPS-II alone. Results show a significant improvement of BRASS as compared to SAPS-II. AIC: Akaike Information Criterion, BIC: Bayesian Information Criterion, NRIc: Net Reclassification Improvement, IDI: Integrated Discrimination Improvement. (DOCX) [file pone.0176012.s006.docx]

**S5 Table. Statistical indexes of the final models.**

|  | **Model** | | |
| --- | --- | --- | --- |
|  | **SAPS-II** | **BRASS** | **SAPS-II and BRASS** |
| **AIC** | 142.3 | 114.3 | 113.4 |
| **BIC** | 148.1 | 120.2 | 122.3 |
| **NRIc *vs* SAPS-II only (SE*)** | - | 0.727 (0.341) | 0.984 (0.200) |
| **IDI *vs* SAPS-II only (SE*)** | - | 0.217 (0.086) | 0.233 (0.077) |

AIC and BIC for models with SAPS-II, BRASS and both, as well as the NRIc and IDI for a model with the BRASS compared to a model with SAPS-II alone. Results show a significant improvement of BRASS as compared to SAPS-II. AIC: Akaike Information Criterion, BIC: Bayesian Information Criterion, NRIc: Net Reclassification Improvement, IDI: Integrated Discrimination Improvement.
