# Supplementary material for: Brainstem response patterns in deeply-sedated critically-ill patients predict 28-day mortality
Source: PLoS One. 2017 Apr 25;12(4):e0176012. doi: 10.1371/journal.pone.0176012 (PMC5404790; doi:10.1371/journal.pone.0176012)
Supplement: S5 Table — Neurological examination was available in 42 out of 69 patients who were alive and had a RASS < - 3 at day 4. Neurological examination was not systematically performed after day 1 in patients with RASS remained below -3. It has to be reminded that brainstem reflexes were never assessed in patients with RASS ≥– 3. All brainstem reflexes were more frequently present on day 4 than on day 1 but also in patients without than with midazolam, except for the cough reflex. Discontinuation of sedation does not imply that there was no persisting effect of midazolam. Mid+: patient receiving continuous midazolam infusion. (DOCX) [file pone.0176012.s007.docx]

| **S6 Table. Brainstem reflexes at day 4 among patients deeply sedated (RASS < -3).** | | | | |
| --- | --- | --- | --- | --- |
| **Responses** | **Day 1** | **Day 4** | **Day 4 Mid+** | **Day 4 Mid-** |
| n | 42 | 42 | 29 | 13 |
| GCS Motor, median (Q1 to Q3) | 1 (1 to 1) | 1 (1 to 3) | 1 (1 to 3) | 1 (1 to 4) |
| GCS Ocular, median (Q1 to Q3) | 1 (1 to 1) | 1 (1 to 2) | 1( 1 to 2) | 1 (1 to 1) |
| Pupillary light reflex, no. (%) | 33 (78.6) | 37 (92.5) | 25/28 (89.3) | 12/12 (100) |
| Corneal reflex, no. (%) | 36 (85.7) | 40 (97.5) | 27/28 (96.4) | 13/13 (100) |
| Oculocephalic reflex, no. (%) | 19 (45.2) | 26 (61.9) | 16/29 (55.2) | 10/13 (77) |
| Grimacing to pain, no. (%) | 22 (52.4) | 27 (67.5) | 17/27 (63.0) | 10/13 (77) |
| Cough reflex, no. (%) | 26 (63.4) | 33 (75.0) | 22/27 (81.5) | 8/13 (61.5) |
| Myosis, no. (%) | 32 (76.2) | 24 (58.5) | 16/28 (57.1) | 8/13 (61.5) |

Neurological examination was available in 42 out of 69 patients who were alive and had a RASS < - 3 at day 4. Neurological examination was not systematically performed after day 1 in patients with RASS remained below -3. It has to be reminded that brainstem reflexes were never assessed in patients with RASS ≥– 3. All brainstem reflexes were more frequently present on day 4 than on day 1 but also in patients without than with midazolam, except for the cough reflex. Discontinuation of sedation does not imply that there was no persisting effect of midazolam. Mid+: patient receiving continuous midazolam infusion.
