# Supplementary material for: Brainstem response patterns in deeply-sedated critically-ill patients predict 28-day mortality
Source: PLoS One. 2017 Apr 25;12(4):e0176012. doi: 10.1371/journal.pone.0176012 (PMC5404790; doi:10.1371/journal.pone.0176012)
Supplement: S1 Appendix — (PDF) [file pone.0176012.s008.pdf]

Patient's ID

Brainstem responses assessment in ICU

Date: (mm/dd/yyyy):														
**Pupils size**	R	L	R	L	R	L	R	L	R	L	R	L	R	L
**Pupillary light reflex**														
**Corneal reflex**														
**Oculocephalic reflex**														
**Grimacing to pain**														
**Cough reflex**														
Remarks														

- Note reflexes as **<u>present</u>** (any reproducible attended movements) or **<u>absent</u>** (0 / 1 or - / +), use right (R) and left (L) when it is appropriate
- **<u>Pupils size</u>** is noted in mm or > < = to 2 mm and 5 mm (if the form is printed in A4 format [1 cm = ⊢—⊣ ; 1 inch = ⊢———⊣]: 2 mm = ● ; 5 mm = ⬤)
- **<u>Corneal reflex</u>** is assessed with eye drop or sterile tissu applied to the sclera and considered present if any contraction of the superior/inferior led or a revulsion of the ocular glob is observed
- **<u>Oculocephalic reflex</u> (OCR)** is considered present if the eyes cross the midline during a lateral passive head rotation (we test the horizontal OCR only)
- **<u>Grimace to pain</u>** is assessed during a bilateral and strong pressure to the retro-mandibular regions
- **<u>Cough reflex</u> i**s assessed in response to a tracheal suctioning and considered positive if any contraction of abdominal muscles is observed

Brainstem Responses Assessment Sedation Score (BRASS)

BRASS is the sum of the following sub-scores:

- absent pupillary light reflex = 1
- absent cough reflex = 1
- absent corneal reflex = 2
- absent grimace to pain <u>and</u> absent OCR = 1
- absent grimace to pain <u>but</u> presence of OCR = 3

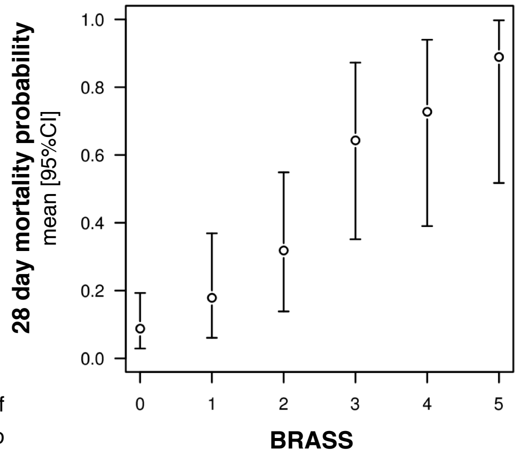

The 28-day mortality predictive value of the BRASS assessed within the first 24 hours of sedation has been developed and validated in deeply sedated patients (no movement to voice; RAAS <-3). Rohaut et al. PLOS ONE 2017.
