# Supplementary material for: Brainstem response patterns in deeply-sedated critically-ill patients predict 28-day mortality
Source: PLoS One. 2017 Apr 25;12(4):e0176012. doi: 10.1371/journal.pone.0176012 (PMC5404790; doi:10.1371/journal.pone.0176012)
Supplement: S2 Appendix — (DOCX) [file pone.0176012.s009.docx]

**S2 Appendix. Details regarding the calibration of the BRASS.**

In order to determine whether modern data mining methods would have led to a more accurate prognostic score than logistic regression, boosted regression trees were also used (1). Three different interaction depths in the regression trees were considered, from one (no interaction) to three, and the model with the best fit was retained. The discrimination of the resulting prognostic score was compared to that of the logistic regression-based score. Since scores obtained by these methods did not provide better discrimination than the simplified score, we do not report on the related results. External validation of the BRASS was performed using the subset of 46 deeply sedated patients from a previous study (2).

The discriminative ability of a model was assessed using the c-index (identical to the area under the receiver operating characteristics [ROC] curve) (3). We compared the capacity for discrimination of different models, using the integrated discrimination improvement (IDI) (4) and the category-less net reclassification improvement (NRIc) (5) indices. Bootstrap standard errors were obtained as this method has been shown to outperform others for NRIc (6).

1. Hastie T, Tibshirani R, Friedman J. Unsupervised learning [Internet]. Springer; 2009 [cited 2015 Sep 4]. Available from: http://link.springer.com/chapter/10.1007/978-0-387-84858-7_14

2. Sharshar T, Porcher R, Siami S, Rohaut B, Bailly-Salin J, Hopkinson NS, et al. Brainstem responses can predict death and delirium in sedated patients in intensive care unit. Crit Care Med. 2011 Aug;39(8):1960–7.

3. Harrell FE, Califf RM, Pryor DB, Lee KL, Rosati RA. Evaluating the yield of medical tests. JAMA. 1982;247(18):2543–2546.

4. Pencina MJ, D’Agostino RB, D’Agostino RB, Vasan RS. Evaluating the added predictive ability of a new marker: from area under the ROC curve to reclassification and beyond. Stat Med. 2008;27(2):157.

5. Pencina MJ, D’Agostino RB Sr, Steyerberg EW. Extensions of net reclassification improvement calculations to measure usefulness of new biomarkers. Stat Med. 2011 Jan 15;30(1):11–21.

6. Kerr KF, Wang Z, Janes H, McClelland RL, Psaty BM, Pepe MS. Net reclassification indices for evaluating risk prediction instruments: a critical review. Epidemiol Camb Mass. 2014 Jan;25(1):114–21.
